# A psychological intervention for caries active young adults, a randomized controlled trial

**DOI:** 10.1002/cre2.513

**Published:** 2021-11-18

**Authors:** Jennie Hagman, Ulla Wide, Helene Werner, Magnus Hakeberg

**Affiliations:** ^1^ Department of Behavioral and Community Dentistry, Institute of Odontology, Sahlgrenska Academy University of Gothenburg Gothenburg Sweden

**Keywords:** behavior therapy, oral health, randomized controlled trial, young adult

## Abstract

**Objective:**

The aim of the present study was to evaluate the effect of a brief version of the behavioral intervention Acceptance and Commitment Therapy (ACT) on reducing gingivitis and plaque levels after 18 weeks.

**Materials and methods:**

One hundred thirty‐five caries‐active young adults (18–25 years of age), recruited from two public dental clinics, participated in this parallel group randomized control trial (RCT). Participants in the intervention (*n* = 67) received two ACT sessions in combination with standard information on oral health, and participants allocated to the control group (*n* = 68) received standard information only. Gingivitis and plaque levels were recorded at baseline and at the 9‐ and 18‐week follow‐ups. The effect of the intervention versus standard information alone was analyzed by intention‐to‐treat and per protocol, applying the General Linear Model (GLM). Exploratory analyses for the intervention and control groups were conducted to evaluate the effect of gender and smoking habits on the gingivitis and plaque outcome. The CONSORT guidelines for RCT were followed.

**Results:**

A significant decrease in gingivitis and plaque levels was observed over time, irrespective of treatment allocation. However, the ACT intervention was not significantly more effective at reducing gingivitis and plaque scores than standard information alone, even though the intervention participants had maintained their improvement to a greater extent. The exploratory analysis revealed that females improved their gingivitis and plaque levels significantly more than the males in the intervention group (*p* = 0.025 for gingivitis and *p* = 0.013 for plaque).

**Conclusion:**

A brief ACT intervention was not proven to be more effective than standard information alone at improving oral health in a sample of young adults with poor oral health. However, ACT seems to have a positive effect on oral health among females. (TRN ISRCTN15009620).

## 1 INTRODUCTION

Oral health behavior and, consequently, the risk of oral disease, is the result of a dynamic interplay between several different influencing factors, both on a more distal level (environment, ethnicity, culture, age, socioeconomic status) and on a proximal level (health beliefs, attitudes, goals) (Morrison & Bennett, 2012). This dynamic interplay calls for interventions on multiple levels (societal, population and individual level) to improve oral health (Smedley & Syme, 2001). Historical behavioral change interventions have mostly targeted risk behavior with the assumption that increased knowledge would lead to long‐lasting improvement in oral health behavior (Daly et al., 2013). This approach has proven ineffective (Daly et al., 2013; Watt & Sheiham, 1999). More recently, the need for interventions based on theories of behavioral change has been emphasized (Kay et al., 2016; Renz et al., 2007). However, there are conflicting opinions regarding the strength of evidence when it comes to the ability of psychological interventions to improve oral health and oral health behavior. While some reviews claim that there is strong evidence (Kay et al., 2016), others adopt a more tentative approach (Renz et al., 2007; Werner et al., 2016). Furthermore, Werner et al. (2016) acknowledge some limitations in previous reports, including the limited generalizability of results to the general population and the lack of studies that include patients with dental caries as well as adolescents and young adults.

Young adults find themselves at a time of their life when they begin making health‐related decisions of their own. Furthermore, it is a time when their health‐related attitudes and behaviors may change (Morrison & Bennett, 2012). This makes young adults a suitable group to target for interventions. Additionally, it is important to prevent oral diseases early due to their degenerative nature. Also, the fact that previous findings suggest that young adults with poor oral health suffer from poor oral health‐related quality of life further stresses the need for effective intervention in this population (Carvalho et al., 2015; Lawrence et al., 2008).

Acceptance and commitment therapy (ACT) is a theory‐based psychological method that has shown positive results in the treatment of addiction, pain and tinnitus (Ruiz, 2012; Powers et al., 2009). Moreover, the possibility to effect behavioral change with only a few sessions and the fact that ACT can be used in primary care settings (Strosahl et al., 2012) makes it a possible method for use in dentistry.

In a previous report from the present randomized control trial (RCT), evaluating the effect of the ACT intervention on oral health behavior, the analysis revealed some important findings (Wide et al., 2018). Thus, the purpose of the present study was to evaluate the effect of the ACT intervention on oral health at the 9‐ and 18‐week follow‐ups. We hypothesized that the ACT intervention would reduce gingivitis and plaque levels more effectively than standard oral health information alone.

## 2 MATERIALS AND METHODS

### 2.1 Study design

A parallel group randomized controlled trial design was applied. The study was conducted at two Public Dental Service clinics (PDS) in Region Västra Götaland, Sweden. Approval for the study was obtained from The Regional Ethical Review Board in Gothenburg (reg. no. 840‐12) and followed The Declaration of Helsinki protocols.

### 2.2 Sample size and participants

A power analysis was conducted to estimate the sample size with gingivitis as the main outcome. A 20% reduction in mean gingivitis between treatment groups was anticipated. The alpha level was set to 0.05 and the power to 0.80. According to the power analysis, 53 individuals were needed per treatment group. Including dropouts, the sample size was set to 65 individuals per treatment group. Additional power analyses were performed for plaque and oral health behaviors, but none of them indicated that a larger sample size was needed to detect significant differences between groups.

**Figure 1:**
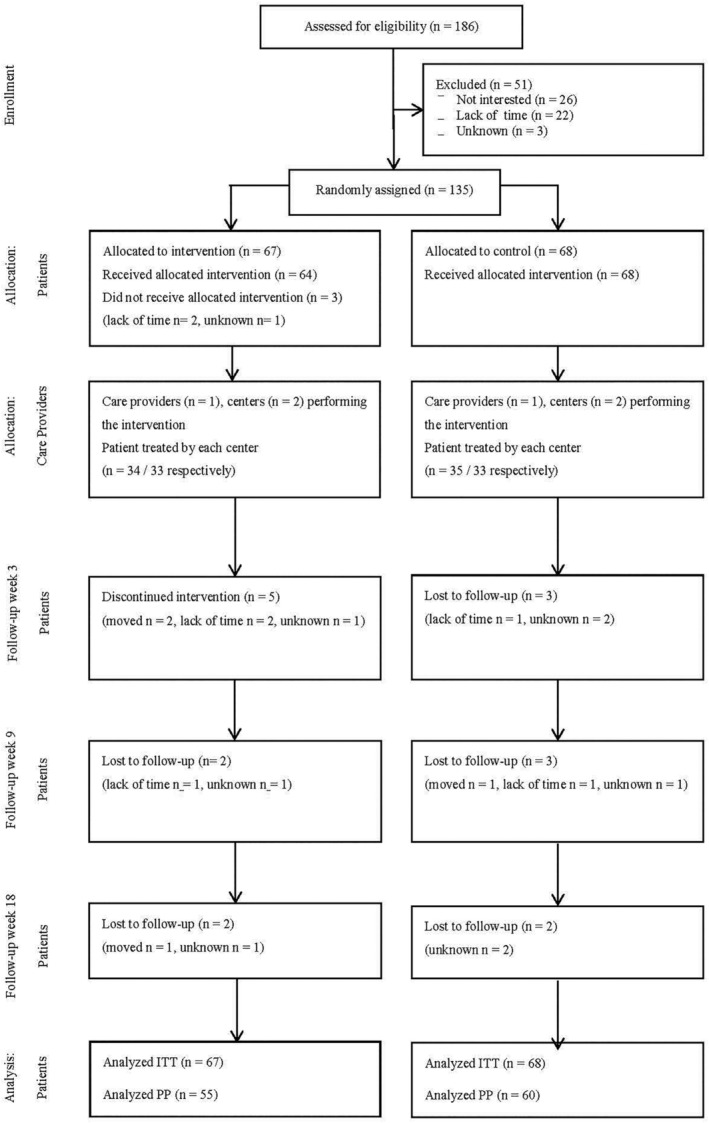
Flow diagram displaying the flow of participants through the various phases and follow‐up events

All young adults attending the PDS clinics between 2013 and 2014 were screened for eligibility in connection with their regular dental check‐up. The inclusion criteria were age 18–25 years and at least two new manifest dental caries lesions on approximal surfaces since the last regular dental examination. The exclusion criteria were a psychiatric/neuropsychiatric diagnosis (i.e., autism spectrum disorder, depression) and the participants needed to have a proper understanding of Swedish. Consecutive eligible patients were asked by their dentist or dental hygienist if they wanted to participate in the trial. If interested, they were contacted by the respective clinic's research coordinator who then confirmed the inclusion/exclusion criteria and gave the participants verbal and written information about the trial. In total, 186 patients were contacted by the research coordinators and of these, 51 declined to participate (see flow diagram, Figure 1). The remaining 135 participants all gave their written and informed consent to participate, before inclusion in the trial.

### 2.3 Procedure and randomization

At baseline, the included participants answered a battery of questions on a touch‐screen computer. In addition, clinical data on plaque and gingivitis were recorded. Data on dental caries were collected from the participants' last ordinary dental examination. All participants then received standard information on oral health, verbally delivered by the research coordinators according to a brochure produced for general use in the PDS clinics in Region Västra Götaland at the time of the trial. The participants were then randomly allocated (allocation ratio 1:1) by the research coordinators to the intervention or control group according to a block randomization procedure, stratified by smoking and gender. The randomization list had been prepared in advance and transferred to sealed opaque envelopes placed in four boxes according to the stratification. The researcher who had prepared the allocation sequence did not take part in the randomization, treatment or data collection procedures and was the only person with access to the allocation key (kept in a locked safety box). Participants allocated to the intervention received two 45‐min ACT sessions. Each session was delivered individually by the same psychologist (HW) at the PDS clinics. The first session was delivered about 2 to 3 weeks after randomization and the second 2 weeks later. Participants were followed up at 3, 9, and 18 weeks after baseline. In the present study, we will be analyzing gingivitis and plaque levels on the 9‐ and 18‐week follow‐up occasions.

### 2.4 Intervention

The psychological intervention was based on Acceptance and Commitment Therapy (Hayes et al., 1999) which is a form of Cognitive Behavioral Therapy (CBT) that aims to promote psychological flexibility and behavioral change so that individuals can live more in accordance with their inner life values. A brief format of ACT already exists, adapted to for example primary care settings (Strosahl et al., 2012). In collaboration with a licensed psychologist specialized in ACT two of the authors (HW, UW) modified this brief format of ACT to fit the aim of the present trial and a dental clinical setting. The psychologist delivering the intervention (HW) was regularly supervised to ensure adherence to the treatment manual. Furthermore, the psychologist (HW) also had solid experience of treating patients with dental phobia and was, therefore, familiar with dentistry and with treating patients in dental settings. For a more detailed description of the intervention, see the previous report from the same RCT (Wide et al., 2018) and the treatment manual (Werner et al., 2020).

### 2.5 Measures

The following variables were analyzed in the present paper:

Sociodemographic characteristics were investigated through questions on gender, age, ethnicity (Swedish‐born, born in another Nordic country or other countries), mother's and father's ethnicity (Swedish‐born, born in another Nordic country or other countries), occupation (employed, unemployed, student), and mother's and father's educational level (primary, secondary school, university).

Oral health behavior was captured by dental care attendance (twice a year, once a year, once every second year, less then every second year, only for emergency care) and smoking habits (yes, no).

Gingivitis was measured at baseline and at the 9‐ and 18‐week follow‐ups. It was defined as bleeding on probing of the gingival sulcus (yes, no) (Muhlemann & Son, 1971) and recorded for mesial, buccal, distal and lingual surfaces of index teeth 16, 21, 24, 44, 41, and 36. Thus, 24 sites were recorded per individual and scores ranged between 0 and 24.

Plaque was measured according to Silness‐Löe plaque index system (with a score of 0–3) (Silness & Loe, 1964) at the mesial, buccal, distal and lingual surfaces of index teeth 16, 21, 24, 44, 41, and 36. The plaque score was dichotomized into “absence of visible plaque” (score 0–1) or “presence of visible plaque” (score 2–3). The scores ranged between 0 and 24.

### 2.6 Statistical methods

Descriptive statistics for baseline characteristics were analyzed by frequencies and plaque and gingivitis by the mean, median, and SD at baseline and on the follow‐up occasions. The General Linear Model (GLM) with repeated measures was applied to analyze the impact of the intervention vs. standard treatment on gingival and plaque scores, across the three measuring occasions (baseline, 9 and 18 weeks after baseline). Both intention‐to‐treat (ITT) and per‐protocol (PP) analyses were conducted. The last observation carried forward was applied to missing data due to drop‐out at the 9‐ and 18‐week follow‐ups. Exploratory sub‐analyses were conducted to analyze if gender or smoking habits had any effect on the outcome for the intervention and control groups, respectively.

## 3 RESULTS

The flow diagram in Figure 1 (according to CONSORT [Boutron et al., 2017]) shows the flow of the participants through the two treatment groups. Of the 12 dropouts in the intervention group, seven were males and three reported being smokers. In the control group, four of the eight dropouts were males and six reported being smokers. Baseline characteristics according to treatment allocation are shown in Table 1. The vast majority of participants visited the dentist regularly (87.5% at least every second year) and about one third reported being smokers. About 26% of the participants reported being born abroad, more than 50% had a parent born abroad and almost 30% reported that their mother and father had an educational level of primary school. A slightly higher proportion of the participants in the intervention group than in the control group reported being unemployed (31.3% vs. 14.7%) and their mothers being Swedish‐born (55.2% vs. 39.7%). The participants had a mean number of 16.4 surfaces with recorded bleeding on probing, a mean plaque score of 7.6 and a mean number of 5.6 manifest dental decays.

**Table 1:** Sociodemographic characteristics, oral health behavior, and oral health status at baseline, by treatment allocation and for the total group

Variable, *n* (%) (SD = standard deviation)	Intervention (*n* = 67)	Control (*n* = 68)	Total (*n* = 135)
Gender			
Female	32 (47.8)	32 (47.1)	64 (47.4)
Age in years, mean (SD)	20.4 (2.1)	20.8 (2.2)	20.6 (2.2)
Dental care attendance			
Two times per year	41 (61.2)	39 (57.4)	80 (59.3)
One time per year	17 (25.4)	17 (25.0)	34 (25.2)
One every second year	2 (3.0)	2 (2.9)	4 (3.0)
Less than every second year	4 (6.0)	6 (8.8)	10 (7.4)
Only for emergency care	3 (4.5)	4 (5.9)	7 (5.2)
Smoking	23 (34.3)	24 (35.3)	47 (34.8)
Ethnicity (Swedish‐born)	52 (77.6)	48 (70.6)	100 (74.1)
Mother's ethnicity (Swedish‐born)	37 (55.2)	27 (39.7)	64 (47.4)
Father's ethnicity (Swedish‐born)	33 (49.3)	29 (42.6)	62 (45.9)
Occupation			
Employed	10 (14.9)	21 (30.9)	31 (23.0)
Unemployed	21 (31.3)	10 (14.7)	31 (23.0)
Student	36 (53.7)	37 (54.4)	73 (54.1)
Mother's education			
Primary	15 (22.4)	22 (32.4)	37 (27.4)
Secondary	35 (52.2)	31 (45.6)	66 (48.9)
University	17 (25.4)	15 (22.1)	32 (23.7)
Father's education			
Primary	17 (25.4)	23 (33.8)	40 (29.6)
Secondary	37 (55.2)	31 (45.6)	68 (50.4)
University	13 (19.4)	14 (20.6)	27 (20.0)
Dental caries, mean (SD) median	6.3 (5.2) 5.0	4.9 (3.7) 4.0	5.6 (4.6) 4.0
Gingivitis, mean (SD) median	16.9 (6.6) 18.0	15.9 (7.1) 16.5	16.4 (6.9) 17.0
Dental plaque, mean (SD) median	7.9 (6.5) 6.0	7.3 (6.4) 6.0	7.6 (6.4) 6.0

Table 2 presents the gingivitis and plaque scores for the intervention and control groups, according to ITT and PP, on all measuring occasions.

**Table 2:** Changes in gingivitis and plaque scores by treatment allocation, presented as mean, standard deviation (SD), median, at baseline and follow‐up 9 and 18 weeks from baseline, according to intention‐to‐treat (ITT) and per protocol (PP) analyses

Variable	Baseline				9‐week follow‐up				18‐week follow‐up			
	Intervention		Control		Intervention		Control		Intervention		Control	
	ITT (*n* = 67)	PP (*n* = 55)	ITT (*n* = 68)	PP (*n* = 60)	ITT (*n* = 67)	PP (*n* = 55)	ITT (*n* = 68)	PP (*n* = 60)	ITT (*n* = 67)	PP (*n* = 55)	ITT (*n* = 68)	PP (*n* = 60)
Gingivitis	16.9 (6.6) 18.0	17.3 (6.6) 18.0	15.9 (7.1) 16.5	15.9 (7.0) 16.0	12.8 (6.4) 13.0	12.5 (6.2) 13.0	12.6 (7.5) 12.0	12.0 (7.2) 11.5	13.3 (7.4) 14.0	13.2 (7.5) 14.0	14.5 (6.6) 15.5	14.2 (6.3) 15.0
Plaque	7.9 (6.5) 6.0	8.6 (6.8) 7.0	7.3 (6.4) 6.0	7.3 (5.9) 6.0	4.2 (5.3) 2.0	4.0 (5.5) 1.0	4.2 (5.7) 2.0	3.8 (4.9) 2.0	4.9 (6.1) 2.0	4.8 (6.5) 2.0	5.5 (6.1) 3.0	5.3 (5.5) 3.0

Intention‐to‐treat analyses with GLM revealed a statistically significant reduction in gingivitis (*p* < 0.001, partial *η* ^2^ = 0.22) and plaque scores (*p* < 0.001, partial *η* ^2^ = 0.28) for both the intervention and the control group over time. The intervention group had maintained their improvement to a greater extent at the 18‐week follow‐up (Table 2). However, the intervention was not statistically significantly more effective than standard information alone at reducing gingivitis or plaque scores. Per‐protocol analyses revealed similar results as the ITT analyses (data not shown).

### 3.1 Exploratory analyses

The separate exploratory analyses of the intervention and control group were conducted to examine whether any difference in treatment efficacy was evident due to gender or smoking behavior.

**Figure 2:**
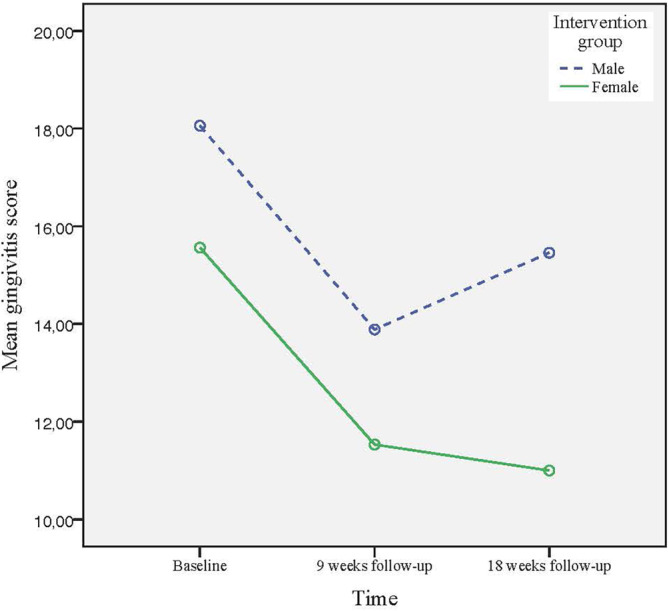
Changes in mean gingivitis score for the intervention group from baseline to the 18‐week follow‐up, according to intention‐to‐treat analysis, for females and males, respectively

**Figure 3:**
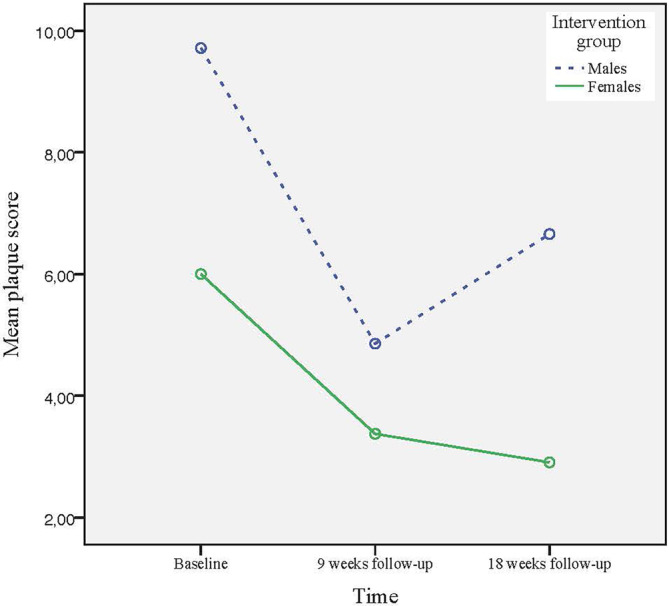
Changes in mean plaque score for the intervention group from baseline to the 18‐week follow‐up, according to intention‐to‐treat analysis, for females and males, respectively

The GLM analysis for gender, according to ITT, demonstrated a statistically significant effect over time, for both genders, at reducing gingivitis (intervention: *p* < 0.001, partial *η* ^2^ = 0.30, control: *p* = 0.002, partial *η* ^2^ = 0.18) and plaque (intervention: *p* < 0.001, partial *η* ^2^ = 0.35, control: *p* < 0.001, partial *η* ^2^ = 0.24), irrespective of which treatment group they belonged to. Moreover, analysis of the intervention group revealed a statistically significant difference in gingivitis and plaque scores at the 18‐week follow‐up, in favor of the females (*p* = 0.025, partial *η* ^2^ = 0.08 for gingivitis and *p* = 0.013, partial *η* ^2^ = 0.09 for plaque) (Figures 2 and 3). This was not the case for the control group, where relapses in gingivitis and plaque scores were obvious at the 18‐week follow‐up for both men and women.

The GLM analysis of smoking behavior, according to ITT, revealed a statistically significant effect of time at reducing gingivitis (intervention: *p* < 0.001, partial *η* ^2^ = 0.32 and control: *p* = 0.008, partial *η* ^2^ = 0.14) and plaque scores (intervention: *p* < 0.001, partial *η* ^2^ = 0.34 and control: *p* < 0.001, partial *η* ^2^ = 0.23), irrespective of treatment group. However, no statistically significant difference in gingivitis or plaque scores was found between different smoking behavior, in either the intervention or the control group. The results of the same exploratory analyses according to PP did not differ considerably from the ITT analyses (data not shown).

None of the participants reported any adverse effects of the intervention.

## 4 DISCUSSION

The present study evaluating an intervention for better oral health revealed a modest reduction in gingivitis and plaque levels among a sample of young adults with poor oral health. Although the reduction was greater for the intervention group, the differences were not statistically significant. However, exploratory analyses revealed a promising effect of the ACT intervention on reducing gingivitis and plaque levels among females receiving the intervention.

Previous research reporting significant effects of theory‐based interventions has mainly focused on psychological interventions to alter specific oral hygiene behaviors in adult patients with periodontal disease, in the context of specialist settings (Godard et al., 2011; Jönsson et al., 2009; Kakudate et al., 2009; Philippot et al., 2005). However, the present sample consisted of young adults with severe caries disease attending public dental clinics, which, to our knowledge, has not been investigated previously; thus, there is a lack of knowledge about this population.

The aim of the ACT intervention was to improve psychological flexibility and to change oral health related behaviors in line with the individuals' inner oral and general health related values (Werner et al., 2020). Increased psychological flexibility, in the present intervention, may enable the individuals to change their oral health‐related risk behaviors (e.g., frequent consumption of sugar‐containing sodas, brushing teeth less than once a day) and thereby live more in line with their oral health‐related life values (such as having whole teeth and a fresh breath when kissing loved ones or applying for a new job). During the ACT session, the psychologist asked the participants if there was any behavior that they would like to alter to improve their oral health. Accordingly, several different oral health‐related behaviors may have been altered; that is, also behavior that would not result in decreased levels of gingivitis and plaque. This may have made it more difficult to detect a significant difference between treatment groups in the present report compared with some of the previous studies on the effect of psychological interventions on oral health.

Furthermore, the short follow‐up times in Kakudate et al. (2009), Philippot et al. (2005), and Godard et al. (2011), and the substantial behavioral reinforcement in Kakudate et al. (2009) and Jönsson et al. (2009) may also have contributed to the different results, compared with the present study.

In the present study, an initial decrease in gingival and plaque levels was observed at the 9‐week follow‐up for both the intervention and the control group. However, at 18 weeks, an increase in the respective levels was evident for both groups, although more pronounced in the control group. These results may indicate that, for some individuals, the initial effect of the ACT intervention is difficult to maintain, or that two ACT sessions may not be enough to produce meaningful change, thus raising the question of the number of sessions needed to achieve and/or maintain behavioral change. Furthermore, Lambert stated that the number of sessions should be guided by the individual treatment response (Lambert, 2007). Hence, there may be a need for more ACT sessions or a booster session to maintain the initial behavior change. Other studies in the literature show a non‐significant outcome of theory‐based behavioral interventions (Brand et al., 2013; Stenman et al., 2012). Like the present study, they applied brief interventions independent of the conventional treatment, and like the present report, they raised the question of the number of sessions needed to achieve behavioral change.

As mentioned, reductions in gingivitis and plaque scores were also evident in the control group. According to Finniss et al. (2010), the treatment response can be attributed to the treatment content and the context, including the psychosocial aspects of the treatment (Finniss et al., 2010). Although oral health information may increase oral health knowledge, it is a less effective strategy for improving oral health (Kay et al., 2016; Satur et al., 2010; Yevlahova & Satur, 2009). It seems reasonable, therefore, to assume that the improvement observed in the control group is a result of an interaction between individual expectations from participating in a trial to promote oral health, conditioning (i.e., association between treatment and improvement), the additional meetings with clinicians, and not wanting to disappoint the clinician (i.e., the participants knew that their oral health status would be evaluated during the follow‐up) (Finniss et al., 2010; Hesser et al., 2011), rather than a result of the oral health information given. Evidently the same argument may be partly true for the intervention group, with regard to the effect of the given oral health information.

An exploratory analysis revealed promising behavioral change among females receiving the ACT intervention, as females decreased their gingivitis and plaque scores statistically significantly more than males in the intervention group. This tendency was not present in the control group. This improvement in oral health most possibly reflected enhanced oral hygiene procedures among the female participants in the intervention group. Male gender has been associated with more unfavorable perceptions, beliefs and attitudes towards oral health and oral health behavior in previous studies (Broadbent et al., 2006; Ericsson et al., 2012; Ostberg et al., 1999). Furthermore, in a qualitative study among adolescents, females were considered, by both genders, to value health more than males (Ostberg, 2005). Although these differences between males and females are most likely the result of psychosocial aspects related to gender norms (Heise et al., 2019; Morrison & Bennett, 2012; Weber et al., 2019), they may also result in a disadvantage for males when it comes to maintaining behavioral change. Another perspective may be that the psychologist providing the intervention was a woman, and thus female participants could respond better to the intervention. Hence, it is possible that men would have benefited from an additional booster session or needed extra sessions to achieve/maintain behavioral change. Moreover, to our knowledge, it is less common in the dental literature to analyze the effectiveness of both the intervention and the control treatment separately in relation to gender and smoking habits. Thus, further research should explore whether gender is of importance to the effectiveness of psychological interventions in dentistry and seek ways to handle these differences.

Unexpectedly, we did not find any associations between smoking behavior and the outcome gingivitis and plaque. The literature suggests that smoking may be a hinder for improving health behaviors (Chassin et al., 1996). However, in the present study, the smokers reduced their amount of gingivitis and plaque over time in concordance with the non‐smoking group. Similar results were found in an RCT study testing an oral health promotion program on smokers (McClure et al., 2020).

There are some weaknesses and strengths to the present study. Due to the wide range of behaviors that could potentially be altered, we may have overestimated the effect size in the power calculations, thereby reducing the chance of detecting significant differences. Moreover, blinding to participants, care providers and those assessing the outcomes was not possible due to the nature of the intervention, which entails a risk of biased results. The drop‐out rate was only 15% at the 18‐week follow‐up. Considering that the sample consisted of young adults, this is less than expected. A higher drop‐out rate has been shown in younger age groups (Swift & Greenberg, 2012). Thus, treatment acceptability was high. Furthermore, the data were analyzed both according to ITT and PP, which has been advised as it provides more information regarding treatment effects under different circumstances (Armijo‐Olivo et al., 2009). The intervention followed a treatment manual, which makes replication and critical evaluation of the intervention possible (Werner et al., 2020). Finally, the external validity could be considered as high, as the intervention was delivered in general dental clinics to a rather large sample of young adults attending the public dental service.

Several systematic reviews emphasize the need to study the long‐term effects of psychological interventions in the field of dentistry (Carra et al., 2020; Järvinen et al., 2018; Renz et al., 2007; Werner et al., 2016). In the present report, approximately 16 weeks had passed since the last ACT session and 9 weeks since the last follow‐up visit, a much longer time than in most previous research. Nevertheless, more time need to have passed if we are going to be able to evaluate fully the efficacy of theory‐based interventions. Furthermore, there is a need for replication of studies on preventive and intervention evaluations to be able to confirm and strengthen the generalizability of the existing results (Beelmann et al., 2018).

## 5 CONCLUSION

A brief intervention based on ACT in a sample of young adults with poor oral health was not more effective than standard oral health information alone in promoting oral health with regard to gingivitis and plaque levels at the 18‐week follow‐up. However, the analysis revealed promising effects of the intervention on oral health among the female study participants, and future studies should test the effect of a booster session.

## CONFLICT OF INTEREST

The authors declare no conflict of interest.

## AUTHOR CONTRIBUTIONS

Magnus Hakeberg and Ulla Wide designed the study. Magnus Hakeberg and Jennie Hagman planned and performed the statistical analyses. Ulla Wide organized the adaptation of the ACT intervention in collaboration with Helene Werner. Helene Werner performed the intervention. Jennie Hagman drafted the manuscript. All authors participated in finalizing the manuscript. All authors gave their final approval and agreed to be accountable for all aspects of the work.

## Data Availability

Due to ethical considerations, the data used for the present study is not publicly available. However, upon reasonable request, the data is available from the corresponding author.
